# A Scanning Microwave Microscopy Study of FIB-Induced Local S_11_ Response Changes in InGaAs/InP and HfO_2_/InGaAs/InP Heterostructures

**DOI:** 10.3390/nano16161042

**Published:** 2026-08-21

**Authors:** Raffaella Polito, Valentina Mussi, Andrea Notargiacomo, Adel Bousseksou, Gregoire Beaudoin, Isabelle Sagnes, Daniele De Felicis, Antonio Valletta, Francesco Mattioli, Edoardo Bemporad, Raffaele Colombelli, Michele Ortolani, Cristian Ciracì, Valeria Giliberti, Marialilia Pea

**Affiliations:** 1 Istituto di Fotonica e Nanotecnologie, Consiglio Nazionale delle Ricerche, 00133 Rome, Italy; raffaella.polito@cnr.it (R.P.); francesco.mattioli@cnr.it (F.M.); michele.ortolani@uniroma1.it (M.O.); marialilia.pea@cnr.it (M.P.); 2 Istituto per la Microelettronica e Microsistemi, Consiglio Nazionale delle Ricerche, 00133 Rome, Italy; valentina.mussi@cnr.it (V.M.); antonio.valletta@cnr.it (A.V.); 3 Centre de Nanosciences et de Nanotechnologies (C2N), CNRS UMR 9001, Université Paris-Saclay, 91120 Palaiseau, France; adel.bousseksou@c2n.upsaclay.fr (A.B.); gregoire.beaudoin@c2n.upsaclay.fr (G.B.); isabelle.sagnes@c2n.upsaclay.fr (I.S.); raffaele.colombelli@c2n.upsaclay.fr (R.C.); 4 Dipartimento di Ingegneria Civile, Informatica e delle Tecnologie Aeronautiche, Università degli Studi Roma TRE, 00146 Rome, Italy; daniele.defelicis@uniroma3.it (D.D.F.); edoardo.bemporad@uniroma3.it (E.B.); 5 Dipartimento di Fisica, Sapienza Università di Roma, 00185 Rome, Italy; 6 Neurophos Inc., 4120 Freidrich Ln, Austin, TX 78744, USA; cristian@neurophos.com; 7 Center for Life Nano- and Neuro-Science, Istituto Italiano di Tecnologia, 00161 Rome, Italy; valeria.giliberti@iit.it

**Keywords:** focused ion beam (FIB) milling, scanning microwave microscopy (SMM), InGaAs/InP heterostructures, HfO_2_ dielectric, nanoscale electrical and dielectric damage, mid-infrared plasmonics, AFM and Raman correlative analysis

## Abstract

In this work, scanning microwave microscopy (SMM) is used to monitor the evolution of the local microwave response, in terms of variations in the S_11_ input reflection coefficient, in two III–V heterostructures relevant to mid-infrared photonics, namely a 150 nm thick heavily doped InGaAs layer on InP and HfO_2_ (35 nm)/InGaAs (150 nm)/InP, subjected to Ga^+^ FIB milling over a broad dose range. By correlating raw, uncalibrated two-dimensional S_11_ maps with atomic force microscopy (AFM) and Raman spectroscopy, we observe a dose-dependent evolution from implantation-dominated behavior to progressive amorphization, layer thinning and surface roughening. In the uncapped InGaAs/InP system, the SMM response varies monotonically with ion dose, consistent with progressive FIB-induced modification of the exposed InGaAs layer and, at larger milling depths, of the underlying InP substrate. In the HfO_2_-capped structure, the microwave response is more complex: the oxide initially acts as a partial buffer against ion penetration, delaying damage transfer, but this effect progressively weakens as the cap is thinned and structurally degraded. The resulting S_11_ contrast may reflect the combined effects of FIB-induced structural modifications, layer removal, local material composition, and surface morphology. Overall, the combined dataset indicates that SMM is a potentially highly sensitive probe of FIB-induced nanoscale modifications in the local microwave response, especially at low doses, provided that suitable on-chip calibration and de-embedding structures are available.

## 1. Introduction

Heavily n-doped semiconductors, including InGaAs, have attracted significant interest for mid-infrared (mid-IR) plasmonic and photonic applications because their high free-carrier density enables strong plasmonic nonlinearities and relatively low-loss waveguiding in compact all-semiconductor integrated platforms [1,2,3]. These properties make InGaAs-based heterostructures on InP substrates promising candidates for on-chip mid-IR sources, modulators, and detectors, especially when integrated with metal gates and high-*k* dielectrics such as HfO_2_ [4], to exploit electro-optic effects through electrostatic modulation of the carrier density in the InGaAs layer [5].

In addition to resist-based lithography approaches for device fabrication, maskless and direct-write micro/nano-fabrication techniques such as focused ion beam (FIB) milling [6] are particularly attractive for the rapid prototyping of novel photonic devices with nanoscale precision and may represent a powerful route for structuring complex III–V heterostructures, including InGaAs-based stacks. However, FIB milling with Ga^+^ ions inevitably induces structural damage, including amorphization, Ga implantation, and selective sputtering, even at relatively low doses outside the milling patterns because of beam-tail effects [7,8,9,10]. Previous studies on III–V semiconductors have documented amorphized sidewall layers in InP, InAs, and GaAs, with thicknesses exceeding those predicted by simple implantation models owing to dynamic damage accumulation [11], as well as damage layer thicknesses on the bottom of the milled region greater than those calculated from theoretical models of ion implantation due to passage of the energetic ion beams through already damaged layers [10]. In InP, recoil-induced phosphorus disorder introduces additional defects, whereas GaAs generally does not exhibit recrystallized phases under comparable conditions [10]. In heavily doped InGaAsP quantum wells and InP-based structures, FIB milling has also been associated with Ga segregation and metallic cluster formation [12,13,14], while increased optical losses arising from implantation and local heating have been evidenced in GaAs [15]. More generally, high-energy ion irradiation can generate deep levels that alter material properties, including reduced carrier mobility [16], as well as changes in dielectric constant variations from defect-induced permittivity changes [17]. Notably, few reports address heavily doped InGaAs or HfO_2_-capped variants on InP, leaving a gap in understanding nanoscale electrical/dielectric gradients critical for plasmonic performance.

Therefore, focused ion beam treatment is expected to alter the local electrical and dielectric properties over shallow and intermediate depths, which are highly relevant for the operation of plasmonic heterostructures. Scanning microwave microscopy (SMM) is particularly suitable for probing such modifications because it is a non-destructive scanning probe technique that provides nanoscale access to the local electrical response related to the complex impedance of the material through the microwave reflection coefficient S_11_ [18,19,20]. SMM is sensitive to both capacitive and resistive contributions and can be used to map local variations in conductivity, permittivity, and carrier density with sub-surface sensitivity [21,22]. In contrast to conventional conductive-atomic force microscopy (C-AFM), which is mainly sensitive to tip-sample contact resistance, SMM probes a significantly larger interaction volume and is, therefore, well suited to the investigation of buried interfaces, dielectric overlayers, and multi-layer semiconductor stacks.

Quantitative and semi-quantitative approaches based on calibration and de-embedding of S_11_ data from SMM have further enabled the extraction of capacitance, resistance, resistivity, and dopant concentration in semiconductor systems [23,24,25]. To date, these approaches have been most successful on relatively simple model systems, such as single-semiconductor samples with known implanted dopants [24] and dielectric stacks with well-defined capacitor geometries [26,27]. By contrast, the quantitative characterization of electrical properties in complex multi-layer structures remains quite unexplored and will require more robust and accurate evaluation strategies to enable the extraction of reliable material parameters. Nevertheless, SMM still remains highly relevant for the qualitative characterization of FIB-milled semiconductor heterostructures, including the InGaAs/InP-based systems investigated here, where implantation, amorphization, and compositional changes are expected to generate nanoscale gradients in electrical and dielectric response that cannot be captured by an approach relying solely on a combination of morphological and spectroscopic characterization.

SMM is employed here to investigate changes in the microwave electrical response of III–V materials subjected to Ga^+^ FIB milling, with and without an HfO_2_ dielectric top capping layer. By combining SMM with AFM-based morphological analysis and Raman spectroscopy, we follow the qualitative behavior of S_11_ data, shown as-collected without any calibration, which reflects local impedance variations induced by 30 kV Ga^+^ irradiation in n^+^-InGaAs (150 nm)/InP and HfO_2_ (35 nm)/n^+^-InGaAs (150 nm)/InP heterostructures. These systems are characterized over a wide FIB dose range, from very low doses, corresponding to the implantation/introduction of defects with no apparent morphology variation, to high milling regimes. The results reveal distinct damage mechanisms and responses, including implantation, amorphization, and surface roughening/structuring, which must be carefully considered when optimizing FIB processing, both for nanodevices in general and more specifically, as discussed here, for mid-IR plasmonic structures. They also show that SMM can be highly sensitive to early-stage FIB-induced modifications, even when the topographic changes are still minimal.

Thus, the present work extends previous SMM studies on layered semiconductors/dielectrics, which mostly focused on simpler model systems, to technologically relevant, heavily doped III–V plasmonic heterostructures processed under realistic Ga^+^ FIB conditions. The comparison between dielectric-capped and uncapped stacks further provides a direct view on how insulating layers may reshape FIB-induced electrical and dielectric damage pathways in multi-layer architectures.

## 2. Materials and Methods

### 2.1. Sample Preparation

Semiconducting layers were grown on InP substrates by metalorganic vapor phase epitaxy (MOVPE) in a D180 Turbodisc reactor (Veeco Instruments Inc., Plainview, NY, USA). Two sample structures were investigated: (i) 150 nm thick n^+^ doped InGaAs (*n* = 8.8 × 10^18^ cm^−3^) and (ii) a 35 nm thick HfO_2_ film grown by atomic layer deposition (ALD) on the same 150 nm thick n^+^ doped InGaAs layer. A Helios 5 CX (Thermo Fisher Scientific, Waltham, MA, USA) dual-beam FIB equipped with a Ga^+^ metal ion source was used for FIB patterning at 30 keV, with the beam current ranging from 1 pA to 24 pA under normal incidence. Arrays with areas of 3 × 3 µm^2^ and 4 × 4 µm^2^ were milled on the sample surfaces. The FIB-milled area corresponding to the lowest dose in each array was defined with a rectangular shape. On InGaAs/InP, an FIB dose in the 2.0 × 10^14^–8.2 × 10^16^ ions/cm^2^ range was employed to treat 25 square areas with ions. For the HfO_2_/InGaAs/InP sample, two arrays of 25 square FIB-milled areas were patterned using two different intervals of ion doses: (i) in the first set, the same interval as that for the case of InGaAs/InP sample was used (2.0 × 10^14^–8.2 × 10^16^ ions/cm^2^); and (ii) a second array of 25 areas was milled using a larger ion dose interval (5.6 × 10^12^–3.1 × 10^17^ ions/cm^2^), with the first two areas treated at the same ion dose of 5.6 × 10^12^ ions/cm^2^. The two intervals overlapped and several ion doses were common to both sets. The highest doses in the second range were selected to obtain areas where FIB milling proceeded into the InP layer for at least several hundred nanometers. The electron column of the dual-beam FIB system was used for SEM imaging.

### 2.2. Atomic Force Microscopy—Scanning Microwave Microscopy

AFM morphology measurements were made both in dynamic mode (force constant of ~40 N/m, resonant frequency ~300 kHz) using silicon probes (PPP-NCH, NANOSENSORS, Neuchâtel, Switzerland) with a <10 nm curvature radius and in contact mode using SMM probes (described below) during S_11_ data map collection. The morphology datasets obtained using the two AFM modes were comparable. Topographical profiles across milled areas were obtained by averaging over at least 10 different scan lines centered in the FIB-treated areas.

FIB-milled depths in the ion-treated areas were determined as the difference between the mean AFM height measured within the milled region and that measured on the surrounding unmilled surface. For depth determination and uncertainty estimation, a central 2 µm × 2 µm area of each square was analyzed, excluding a peripheral frame at least 1 µm wide to minimize edge effects. Mean height values and standard deviation were calculated from a 15 × 15-pixel matrix (N = 225), with sampling points separated by at least 100 nm to avoid oversampling of spatially correlated height values. Error bars in the FIB-milled-depth versus ion-dose plots were calculated by combining in quadrature the standard deviations measured within the milled area and on the surrounding unmilled surface.

SMM measurements, i.e., local complex reflection coefficient S_11_ amplitude and phase data maps, were collected using a Nanosurf Flex AFM equipped with a Nanosurf SMM module (Nanosurf AG, Liestal, Switzerland) operating in the range 4.5–6.0 GHz. Details of the specific frequency (which is around 5.5 GHz) used for collecting S_11_ data shown in specific plots are reported in the corresponding figure caption. SMM probes (RMN 25PtIr300B model, Rocky Mountain Nanotechnology, Salt Lake City, UT, USA) have bulk Pt/Ir cantilevers (resonant frequency of ~19 kHz, force constant of ~22 N/m) and tips (curvature radii of ~20 nm). They were employed in contact mode with a set-point force of ~20 nN and scan rate of 0.5 Hz (scan pixel resolutions of 256 × 256 and 512 × 512, scan area from 10 × 10 µm^2^ to 35 × 35 µm^2^). SMM measurements were carried out following the standard procedure for maximizing sensitivity, which consists of tuning the working frequency to the absolute minimum of the S_11_ amplitude sweep versus frequency (instrument range: 4.5–6.0 GHz). This tuning was repeated after each SMM probe change. Data collection was carried out using a 6 dBm power setting and without any DC bias.

No SMM calibration de-embedding procedure was applied; therefore, the reported SMM data consist of uncalibrated S_11_ amplitude and phase signals, which are not converted into absolute impedance, capacitance, permittivity, or conductivity values. On-chip calibration structures would have been required for this purpose. However, fabricating them before FIB processing could have exposed them to ion-induced damage, whereas fabricating them afterwards could have altered the treated regions. The SMM measurements were, therefore, used for qualitative and relative comparison of the local microwave response.

Profiles of the S_11_ amplitude and phase signals across milled areas were obtained by averaging over at least 10 different scan lines centered in the FIB-treated areas. S_11_ amplitude and phase mean and standard deviation values in the milled areas were estimated by analyzing a 2 µm × 2 µm central region in each treated square, excluding a peripheral frame at least 1 µm wide to get rid of edge effects. Mean values and standard deviation were calculated from a 15 × 15-pixel matrix (N = 225). For the InGaAs/InP sample, a smaller area (1.2 µm × 1.2 µm, 10 × 10 pixels, N = 100) was used to estimate S_11_ data mean and standard deviation due to more relevant border effects. The selected pixels were spaced by at least 100 nm to avoid oversampling. Error bars reported for the SMM amplitude and phase versus milled depth correspond to the standard deviation of the S_11_ data distribution within the corresponding FIB-milled area.

### 2.3. Micro-Raman Spectroscopy

Micro-Raman spectra in each FIB-milled area were collected in back-scattering geometry by using a DXR2xi Raman Imaging Microscope (Thermo Fisher Scientific, Waltham, MA, USA). The measurements were performed by exciting the samples at 532 nm with 5 mW laser power and a 50× objective, by using a high-resolution grating with 1800 lines/mm (spectral resolution of about 1 cm^−1^). Each spectrum resulted from 1000 accumulations of 200 ms acquisitions. The spectra were compared after smoothing (Savitzky–Golay filter with a 5-point window), fluorescence background subtraction (polynomial of order 3), and baseline correction.

## 3. Results

### 3.1. Morphological Analysis—SEM and AFM Morphology of FIB-Milled Areas

**Figure 1:**
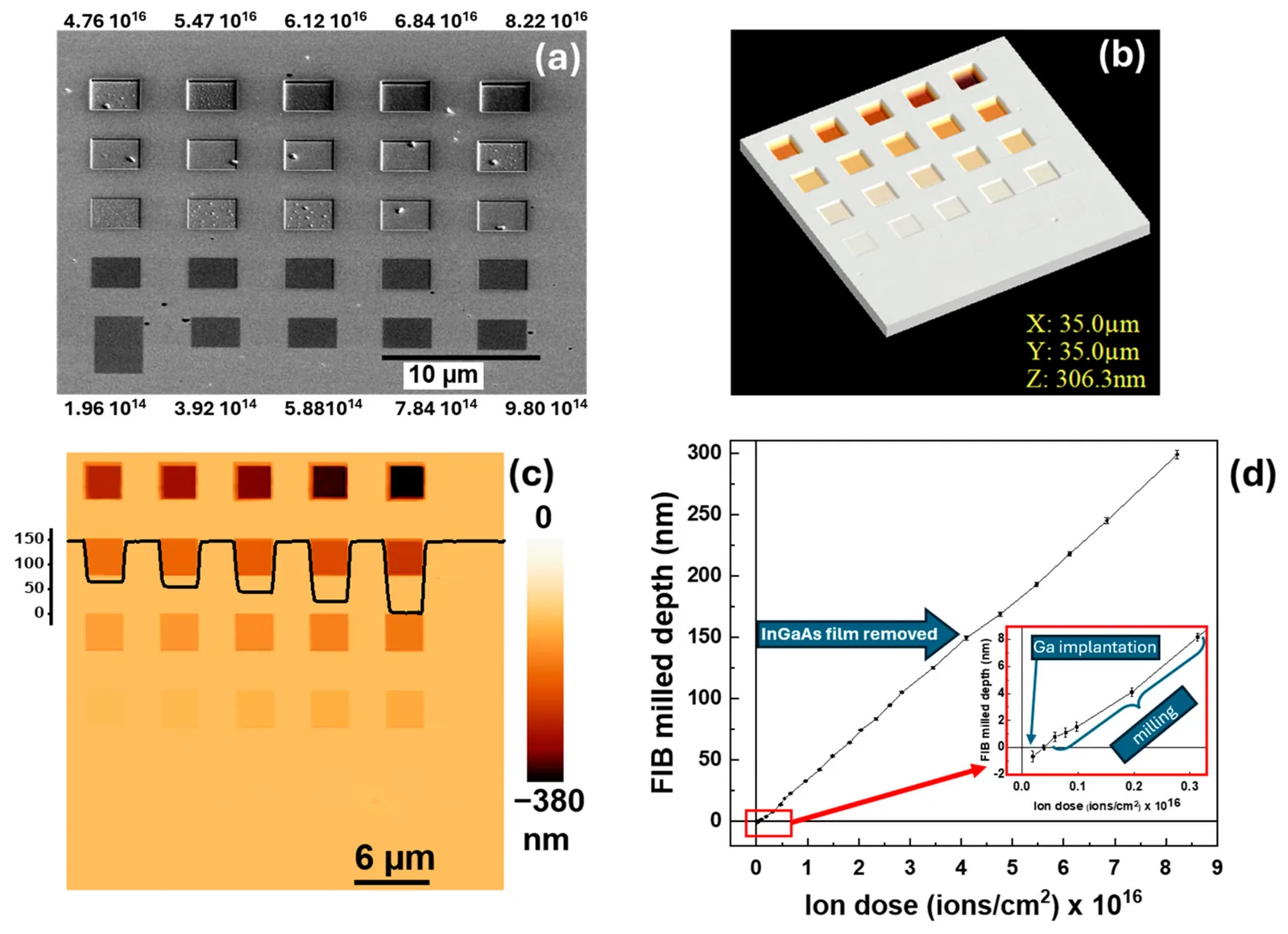
Morphology of the InGaAs/InP sample treated by FIB at increasing ion dose on a 5 × 5 matrix of 3 µm wide square areas. (**a**) SEM micrograph. The two rows of numbers in scientific notation at the bottom and top of the panel indicate the FIB ion doses for the bottom and top rows of treated areas, respectively. (**b**) Three-dimensional view of AFM topography; the reported scale bars correspond to the x, y, and z dimensions of the bounding box containing the 3D rendered surface. (**c**) Two-dimensional AFM map (a section profile of the upper second row of FIB-treated areas is superimposed on the topography map). (**d**) Depth of the FIB-treated areas as measured with AFM. Error bars in the plots represent a combination of the standard deviation of AFM data within corresponding FIB-milled area with the standard deviation in the unmilled areas. The inset (highlighted by a red contour and indicated by the red arrow) shows a magnified view of the low-dose region.

The morphology of the samples was analyzed using SEM and AFM. In the InGaAs/InP sample, the FIB-treated areas exhibit a generally uniform surface (Figure 1a–c). The ion dose range investigated allowed us to produce square pits in the InGaAs film at lower doses and to remove the entire InGaAs and 150 nm of the InP bottom substrate at the highest ion dose. At intermediate doses (1 × 10^16^–5 × 10^16^ ions/cm^2^), the typical formation of metallic gallium droplets is observed, commonly detected on GaAs-based materials irradiated with FIB [12]. The Ga droplets tend to coalesce and then progressively decrease in volume and number, until they disappear upon complete consumption of the InGaAs film. The FIB milling rates on InGaAs and InP are very similar, as evidenced by the nearly linear milling depth versus ion dose curve shown in Figure 1d. A slight decrease in milling rate is observed at ion doses close to the transition between the two materials, likely due to the presence of the interface. AFM measurements show a swelling in the irradiated areas at the lowest ion dose of around 2 × 10^14^–3 × 10^14^ ions/cm^2^, corresponding to a mound ~0.6–0.7 nm thick. The corresponding data point is visible in the inset of Figure 1d as the first experimental point with negative milling depth (i.e., swelling), labeled “Ga implantation.” Such swelling behavior in this very-low-dose regime points to a marked incorporation (implantation) of gallium into the InGaAs when the milling mechanism is not yet fully active, while at slightly higher doses of ~5 × 10^14^ ions/cm^2^ and larger, the milling effect is easily detectable.

It is worth noting that after a few AFM scans in contact mode, even at a force as low as 20 nN, almost all the Ga droplets are moved aside from the scan area, leaving a smooth semiconductor surface, as visible in the AFM maps of Figure 1b,c. SMM investigation was performed after complete removal of the Ga droplet. The surface RMS roughness (Rs) of the FIB-milled area was also measured after these few preliminary scans, showing values below 1 nm for ion doses below ~5 × 10^16^ ions/cm^2^ (corresponding to a milling depth of ~180 nm) and between 1 nm and ~4 nm for doses in the 5 × 10^16^–8 × 10^16^ ions/cm^2^ range.

**Figure 2:**
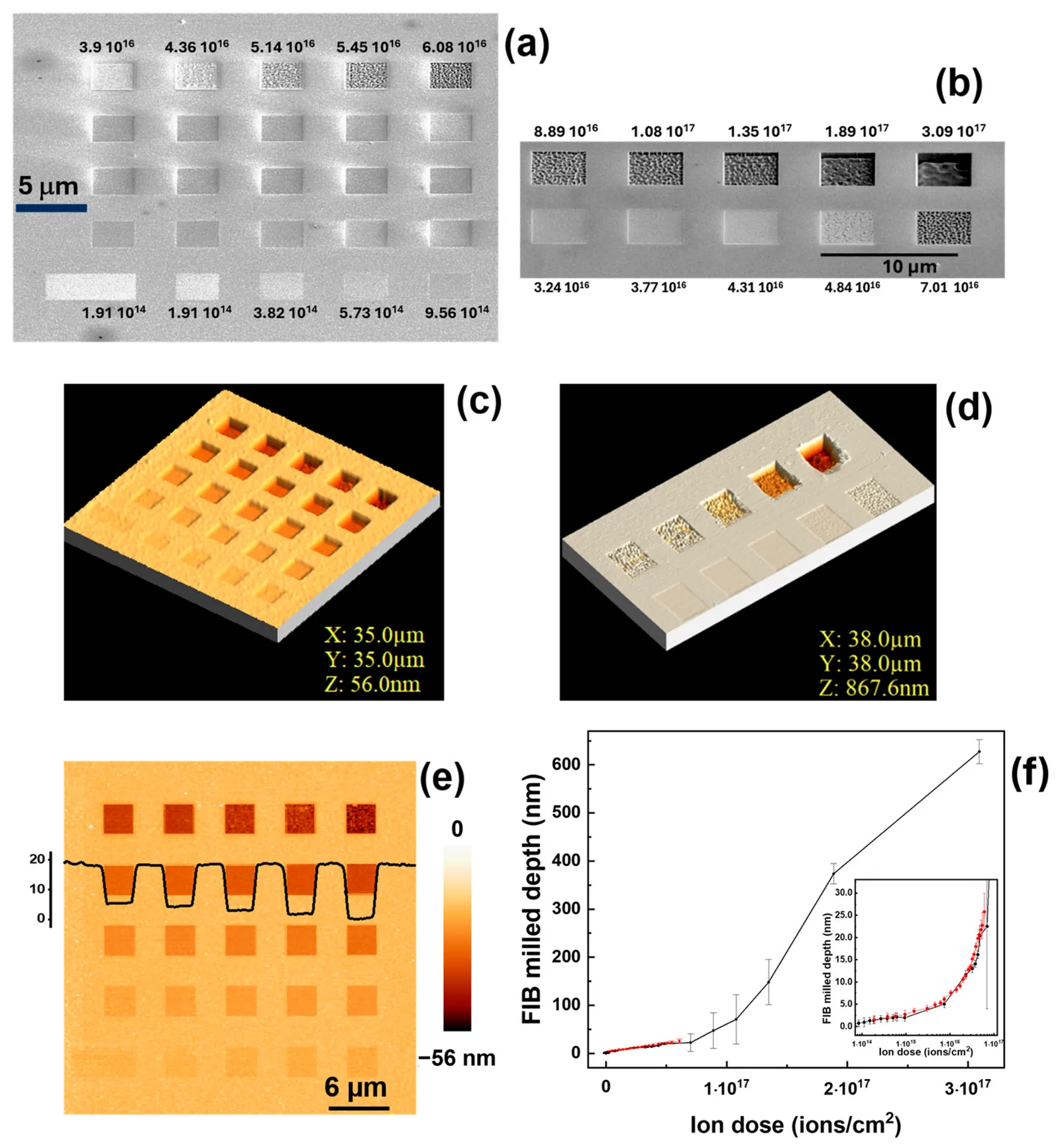
Morphology of the HfO_2_/InGaAs/InP sample treated by FIB. (**a**,**b**) SEM micrographs of area treated with two different dose ranges: (**a**) 1.9 × 10^14^–6.8 × 10^16^ ions/cm^2^; 3 µm wide areas and (**b**) 3.2 × 10^16^–3.1 × 10^17^ ions/cm^2^; 4 µm wide areas. The two rows of numbers in scientific notation at the bottom and top of the panels indicate the FIB ion doses for the bottom and top rows of treated areas, respectively. (**c**,**d**) Three-dimensional view of AFM topographies corresponding to sample areas shown in panels (**a**,**b**). (**e**) Two-dimensional AFM map of sample area shown in panel (**a**); a section profile of the upper second row of FIB-treated areas is superimposed on the topography map. (**f**) Depth of the FIB-treated areas as measured with AFM relative to the small ion dose range (red data points, corresponding to the topographic data of panel (**a**,**c**,**e**)) and to the large ion dose range (black data points), as described in Section 2.1. Error bars in the plots represent standard deviation of AFM data within corresponding FIB-milled area. The reported scale bars in the 3D rendering of AFM data (panels (**c**,**d**)) correspond to the x, y, and z dimensions of the bounding box containing the 3D rendered surface.

FIB milling on the HfO_2_/InGaAs/InP sample was performed by irradiating two 5 × 5 arrays of square areas (3 µm size) with gallium ions at increasing doses in the two ranges described in Section 2.1. The SEM and AFM topography images referring to the array in the dose range of 2.0 × 10^14^–6.1 × 10^16^ ions/cm^2^ are shown in Figure 2a,c,e. The SEM and AFM topography images corresponding to the second treatment range (5.6 × 10^12^–3.1 × 10^17^ ions/cm^2^) are shown in Figure 2b,d, limited to the ten highest doses used. The milling depth measured for the two sets of treated areas is reported in the plot in Figure 2f, showing a substantial overlap of the data and confirming the reproducibility of the FIB milling process.

A pronounced morphological variation is evident at FIB doses corresponding to the milling transition from the HfO_2_ film to the underlying InGaAs film, around a dose of approximately 8.9 × 10^16^ ions/cm^2^. In this transient region, the surface morphology changes from smooth with well-defined profiles for milling depths below 35 nm (corresponding to the HfO_2_ film thickness) to a highly rough character. This occurs due to the inhomogeneity in the removal of the last HfO_2_ layer, where voids are formed non-uniformly. Due to the marked difference in milling rate between HfO_2_ and InGaAs (the ratio of the HfO_2_ milling rate relative to the two III–V materials is approximately 1:12), at these voids, the underlying InGaAs is milled rapidly. This morphological inhomogeneity persists at increasing doses, even after the hafnium oxide film is removed, and leads to a less regular transition between the layers, favoring the coexistence of the HfO_2_ and InGaAs films, and even of the underlying InP film, within the same area at the highest set of ion doses (8 × 10^16^–3 × 10^17^ ions/cm^2^). It is worth noting that small pillars of HfO_2_ are still visible at an ion dose as high as 1.3 × 10^17^ ions/cm^2^, where the surrounding materials have been milled to a depth of approximately 150 nm (Figure 2b). These rough patterns are visible for the five highest doses of the first square array of treated areas (Figure 2a,c,e) and for the seven highest doses of the ten areas shown in Figure 2b,d. At high ion doses, this morphological configuration leads to large experimental uncertainties in the AFM data of FIB-milled areas, with error bars dominated by the high Rs surface roughness (i.e., the standard deviation of height within the treated regions). This behavior is expected because, as mentioned, the milling process does not proceed uniformly from one layer to the next, and the measured data effectively originate from surfaces belonging to different layers.

### 3.2. Structural Analysis—Raman Spectra of FIB-Milled Areas

Raman spectroscopy has been widely used to assess FIB-induced structural damage in III–V semiconductors and heterostructures, as well as in thin oxide films [28,29,30,31]. In general, ion irradiation produces broadening, shifts, and intensity changes in the Raman-active vibrational bands, reflecting disorder, amorphization, implantation damage, strain, and changes in local bonding. These spectral modifications provide a convenient way to estimate the FIB-milling-induced evolution of the structural and chemical properties in the InGaAs, InP, and HfO_2_ materials that are the objects of this work.

**Figure 3:**
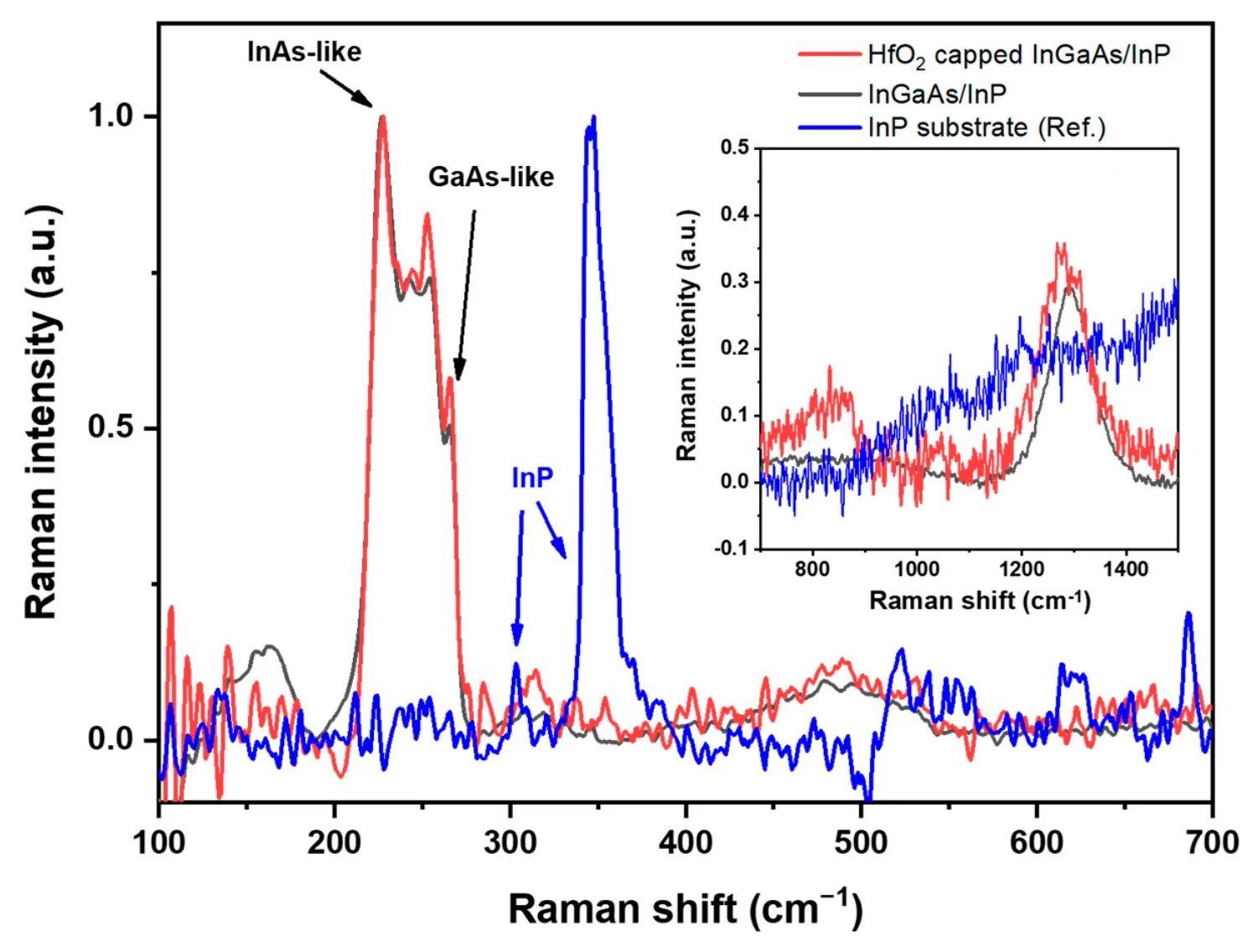
Raman spectra acquired on the untreated regions of the two samples, InGaAs (150 nm)/InP (black curves) and HfO_2_ (35 nm)/InGaAs (150 nm)/InP (red curves), and on the InP bare substrate (blue curves). The inset shows a detailed view of the spectral range 700–1500 cm^−1^.

The Raman spectra of the two samples in the regions not treated with FIB are reported in Figure 3, together with the reference spectrum of the InP material acquired on the bare InP substrate. The effects of potential laser heating are negligible owing to the low laser power and rapid acquisition (200 ms), as confirmed by repeated measurements showing no morphological or spectral changes in the analyzed area. The Raman spectrum of InP shows the characteristic InP-TO (307 cm^−1^) and InP-LO (346 cm^−1^) peaks.

Noticeably, the spectra of the two samples with and without an HfO_2_ film show similar features. Peaks due to the InAs-like LO and GaAs-like LO modes are clearly present, positioned at approximately 228 cm^−1^ and 267 cm^−1^, respectively, which are typical of the ternary InGaAs alloy. The two contributions are not sharply distinct, owing to the overlap with intermediate features associated with various mechanisms, including high doping, strain and possible local disorder [32]. The small differences in the relative intensity of the InGaAs-related peaks are likely due to the HfO_2_ film deposition process and/or interface states in the capped sample.

In the high-wavenumber spectral region (700–1500 cm^−1^, see inset of Figure 3), broad bands are present, at around 1300 cm^−1^, with different intensities for the two samples, commonly attributed to carbon contamination. No other relevant structures ascribable to the semiconducting materials or to the HfO_2_ film are visible, indicating good optical transparency of the capping oxide film at the laser wavelength used.

**Figure 4:**
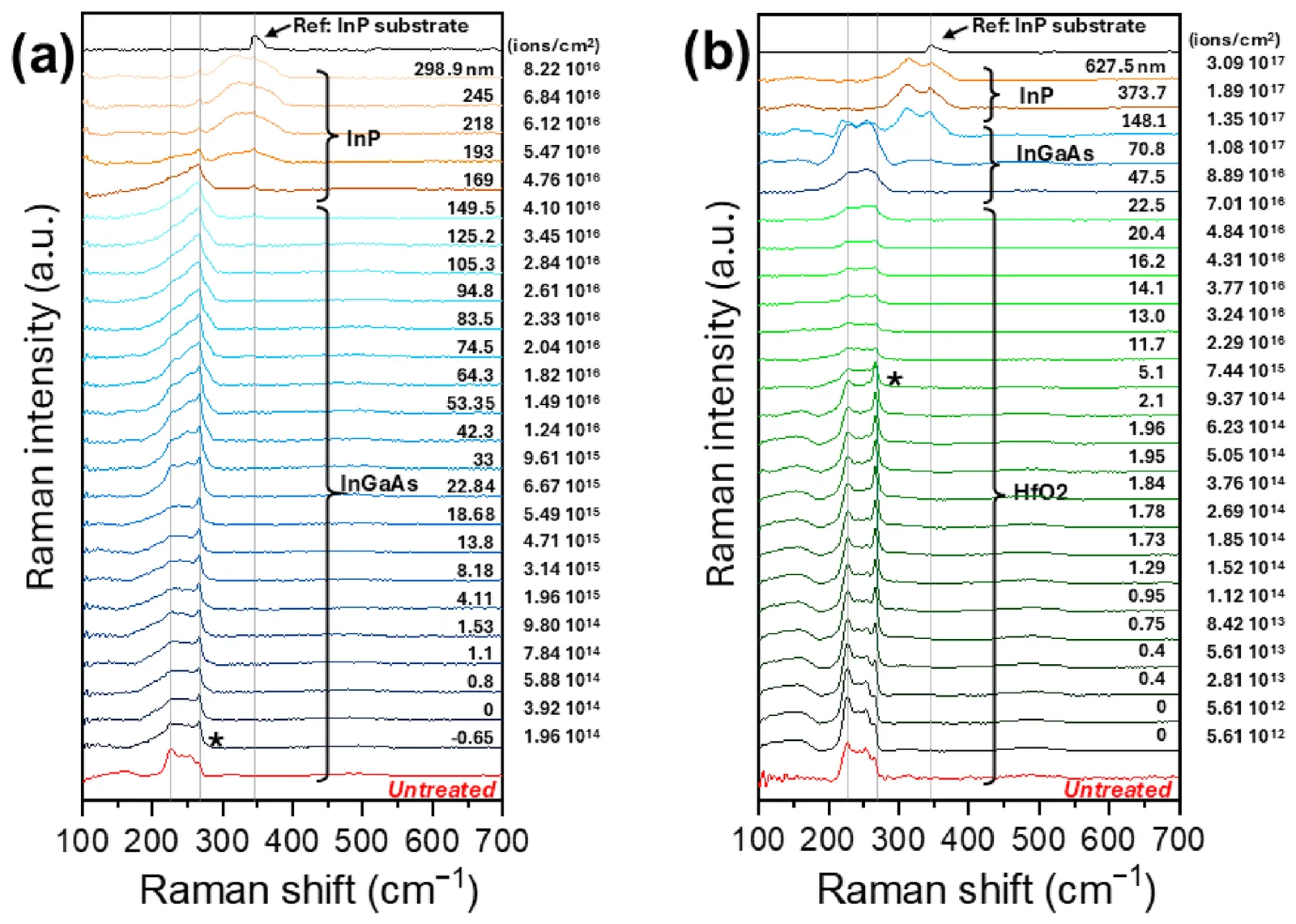
Raman spectra collected in the FIB-treated regions on the two investigated samples: (**a**) InGaAs (150 nm)/InP and (**b**) HfO_2_(35 nm)/InGaAs(150 nm)/InP. The green, blue and brown colours, in their respective shades, indicate the exposed surface layer (HfO_2_, InGaAs or InP) at different milling depths as the stack is progressively milled. The curves marked with an asterisk in the two panels exhibit an almost identical shape and a similar intensity ratio between the InAs-like and GaAs-like peaks. Bottom (red) curves are the spectra corresponding to the respective untreated areas. Top (black) curves show the reference spectrum for InP collected on the bare InP substrate. Vertical gray lines are visual guides corresponding to the main Raman peak positions. Milled depth and ion dose values for each spectrum are reported in each plot, respectively, inside and outside the right side of the frame.

The Raman spectra collected from the regions treated with FIB at increasing doses in the two samples are shown in Figure 4, where right-side labels indicate the FIB dose corresponding to each spectrum while the associated milling depth in nanometers is reported just inside the plots on the right side. Data for the HfO_2_ capped samples are reported for the larger dose range investigated, 5.6 × 10^12^–3.1 × 10^17^ ions/cm^2^.

#### 3.2.1. Raman Spectra of InGaAs/InP Sample

The Raman spectra of the 150 nm thick heavily doped InGaAs layer grown on an InP substrate show a clear evolution as a function of FIB dose. Three distinct regimes can be identified: low, intermediate, and high milling doses. In the low-dose range (~2 × 10^14^–5 × 10^15^ ions/cm^2^), corresponding to an FIB milling depth of up to approximately 15 nm, the most noticeable feature is an abrupt change in the relative intensity of the GaAs-like and InAs-like contributions, with respect to the spectrum of the untreated sample, already at the lowest investigated dose (1.96 × 10^14^ ions/cm^2^).

At this very low dose, the milling depth is barely measurable, while most of the incident Ga^+^ ions are expected to be implanted near the surface, where they form Ga-As-related complexes, leading to an inversion of the InAs-like/GaAs-like peak intensity ratio. In addition, implantation-induced lattice damage causes broadening of InAs-like and GaAs-like Raman bands. As the dose increases, this broadening becomes more pronounced and is accompanied by a progressive reduction in the InAs-like component, consistent with a gradual increase in the local Ga/In ratio within the irradiated regions. In this initial stage, ion implantation dominates over sputtering, causing a modification of the effective composition and a relative enhancement of the GaAs-like contribution with respect to the InAs-like one. The persistence of a stronger GaAs-like peak over the entire investigated dose range indicates a continuous depletion of indium relative to gallium in the semiconductor. The broadening and attenuation of the InGaAs-related band intensity can be attributed to ion-bombardment-induced defects and progressive amorphization of the material. Under these conditions, the milling process evolves from an initially shallow damage regime toward a steady-state condition, in which the shallow defected layer in the InGaAs film is expected to be ~40 nm thick. This assumption for the damage layer thickness is based on experimental measurements of 30 kV Ga^+^ FIB-induced damage in InAs and GaAs layers obtained by TEM reported in previous studies [10]. The adoption of a similar extent for InGaAs is also consistent with SRIM/TRIM simulations, which predict comparable projected ranges for 30 kV Ga ions across all three semiconductors. However, these simulations yield damage depths approximately half of those observed experimentally. This discrepancy arises because SRIM/TRIM does not account for crystal structure or crystallographic orientation, relying primarily on material density. For this reason, in the present work, the thicknesses of the damaged regions are taken as consistent with those reported in Ref. [10], which provides a more realistic estimate of the damage extent. In the intermediate-dose range (~5 × 10^15^–4 × 10^16^ ions/cm^2^), corresponding to a milling depth between ~15 nm and 150 nm, the InAs-like component progressively decreases and develops a strongly asymmetric, almost triangular profile, with a more pronounced tail toward lower wavenumbers. In this regime, weak intermediate features between the two main InAs-like and GaAs-like Raman peaks become visible, particularly in the dose range corresponding to a removed InGaAs depth of approximately 30 nm to 50 nm (~1.5 × 10^16^ ions/cm^2^).

In the highest dose range (4 × 10^16^–6 × 10^16^ ions/cm^2^), corresponding to a removed material thickness of approximately 150–300 nm, a weak but distinct peak centered at about 347 cm^−1^ appears and starts increasing in intensity. This feature can be attributed to the LO Raman mode of InP, indicating that the residual InGaAs layer has become sufficiently thin to allow the probe to sense the underlying substrate. In the same spectral region, a large band arises, progressively broadens, and slightly shifts towards lower wavenumbers with increasing dose, consistent with the accumulation of implantation-induced defects in the crystalline InP and with the development of a steady-state damaged layer, expected to extend to about 60 nm under the employed FIB milling conditions [10].

#### 3.2.2. Raman Spectra of HfO_2_/InGaAs/InP Sample

The Raman spectra of the HfO_2_/InGaAs/InP sample show an evolution qualitatively similar to that observed for the InGaAs/InP sample, with some specific differences associated with the presence of the HfO_2_ top layer. The investigated ion-dose range is broader, spanning approximately 5.6 × 10^12^–3.1 × 10^17^ ions/cm^2^, in order to include both a low-dose regime, in which the FIB effects are largely confined within the 35 nm thick HfO_2_ film, and a high-dose regime, in which the InGaAs layer is completely removed, exposing the underlying InP substrate. With respect to untreated regions, the FIB irradiation induces a more gradual evolution in the intensity ratio between the InAs-like and GaAs-like peaks, which changes from an InAs-like-dominated to a GaAs-like-dominated type. At doses around 5 × 10^14^ ions/cm^2^, corresponding to a reduction in the HfO_2_ thickness by less than 2 nm, the InGaAs-related band begins to broaden symmetrically on both sides, in contrast with the uncapped InGaAs/InP sample. This behavior suggests that some Ga ions penetrate through the HfO_2_ layer and reach the underlying InGaAs film, producing a moderate degree of defectivity. At a dose of ~7 × 10^15^ ions/cm^2^, the Raman features of the InGaAs-related band resemble those of the InGaAs/InP sample irradiated at a dose of about 2 × 10^14^ ions/cm^2^ (see the two corresponding curves marked with an asterisk in Figure 4a,b). This comparison suggests that HfO_2_ initially acts as a buffer layer, retaining most of the incident Ga ions within the oxide until significant amorphization of the cap allows the ions to reach the semiconductor material below. For doses above ~7 × 10^15^ ions/cm^2^, the two main Raman contributions remain comparable in intensity, although the overall InGaAs-related band gradually decreases until the HfO_2_ layer is almost completely removed, which happens at a dose of about 8 × 10^16^ ions/cm^2^ corresponding to an FIB-milled depth of approximately 35 nm, consistent with the nominal oxide layer thickness. As the dose increases further, the InGaAs band broadens and increases in intensity. When the residual InGaAs thickness approaches 35–40 nm, a Raman feature associated with InP becomes visible near 340 cm^−1^. This feature then evolves into two well-defined and intense peaks, typical of crystalline InP, once the InGaAs layer is fully removed at doses around 1.9 × 10^17^ ions/cm^2^, when InGaAs Raman bands disappear.

Overall, the Raman analysis shows a less pronounced broadening of the InGaAs band, accompanied by a smaller loss of the InAs-like component, for the sample with the HfO_2_ top layer. This result is consistent with a protective or “ion-shielding” effect of the dielectric layer in the HfO_2_/InGaAs/InP sample. For both samples, once material removal reaches the InP substrate, the Raman spectra show large defect bands with superimposed features related to crystalline materials.

### 3.3. SMM Mapping of FIB-Milled Areas

As mentioned, focused ion beam treatment is expected to induce changes in the electrical and dielectric properties of the material over a shallow depth, typically extending to several tens of nanometers. SMM is capable of probing the microwave response of materials through the S_11_ coefficient, i.e., the ratio between the microwave power delivered to the tip and the power reflected back from the tip–sample interface, providing information about local electrical properties, even in non-conductive systems. Moreover, SMM can probe a significantly large interaction volume, enabling subsurface investigation with a probing depth on the order of 100 nm depending on the material under study, substantially exceeding that of conventional conductive AFM, which is mainly sensitive to tip–sample contact resistance. It is, therefore, a method of choice for providing complementary information on subsurface electrical variations in FIB-milled areas.

**Figure 5:**
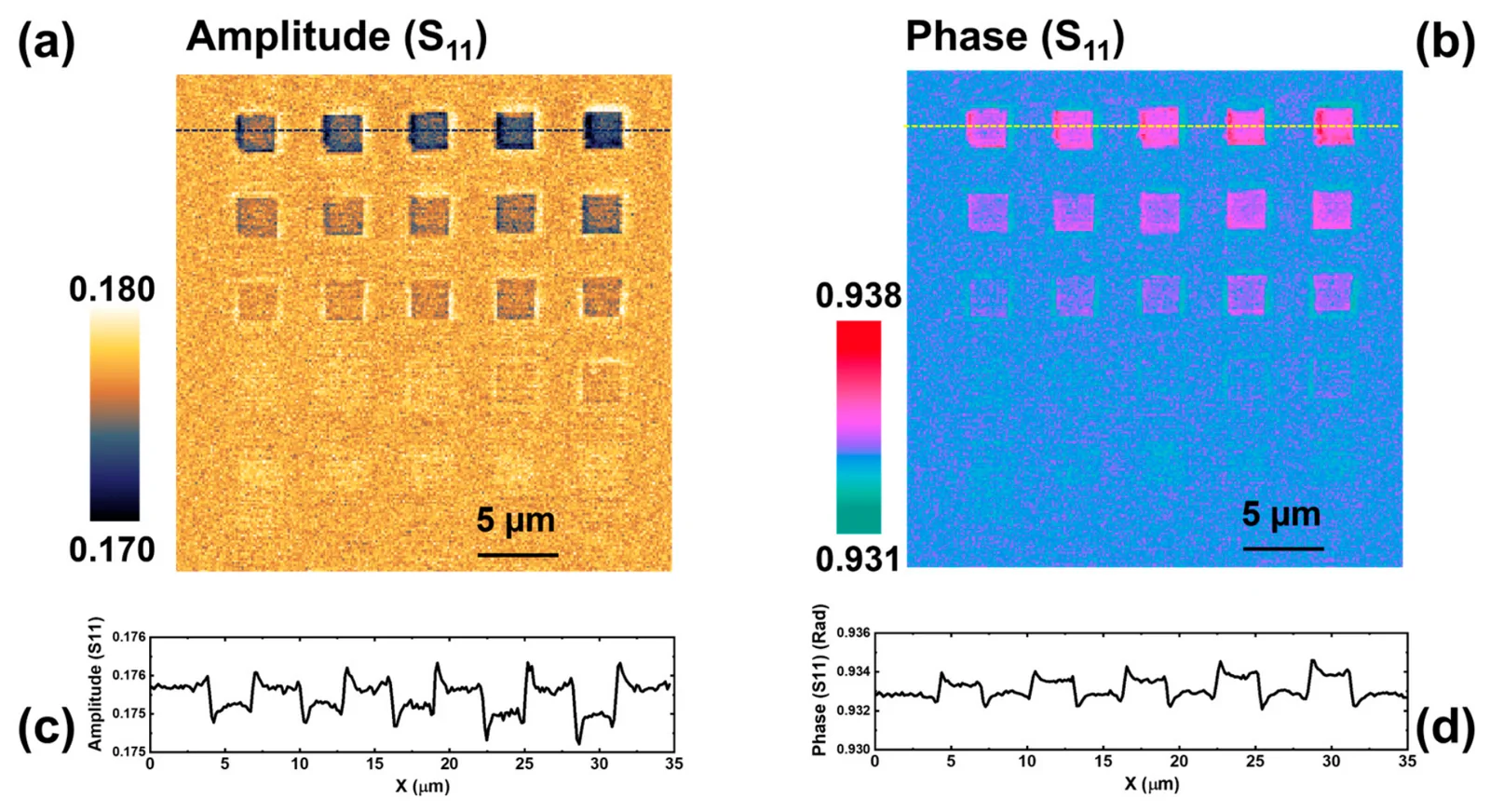
SMM data collected on the InGaAs/InP sample. SMM map of (**a**) amplitude and (**b**) phase of S_11_. Section profiles of (**c**) amplitude and (**d**) phase of S_11_ along the dashed lines shown in (**a**,**b**), respectively. SMM maps were collected at a frequency *f* = 5.484258 GHz.

**Figure 6:**
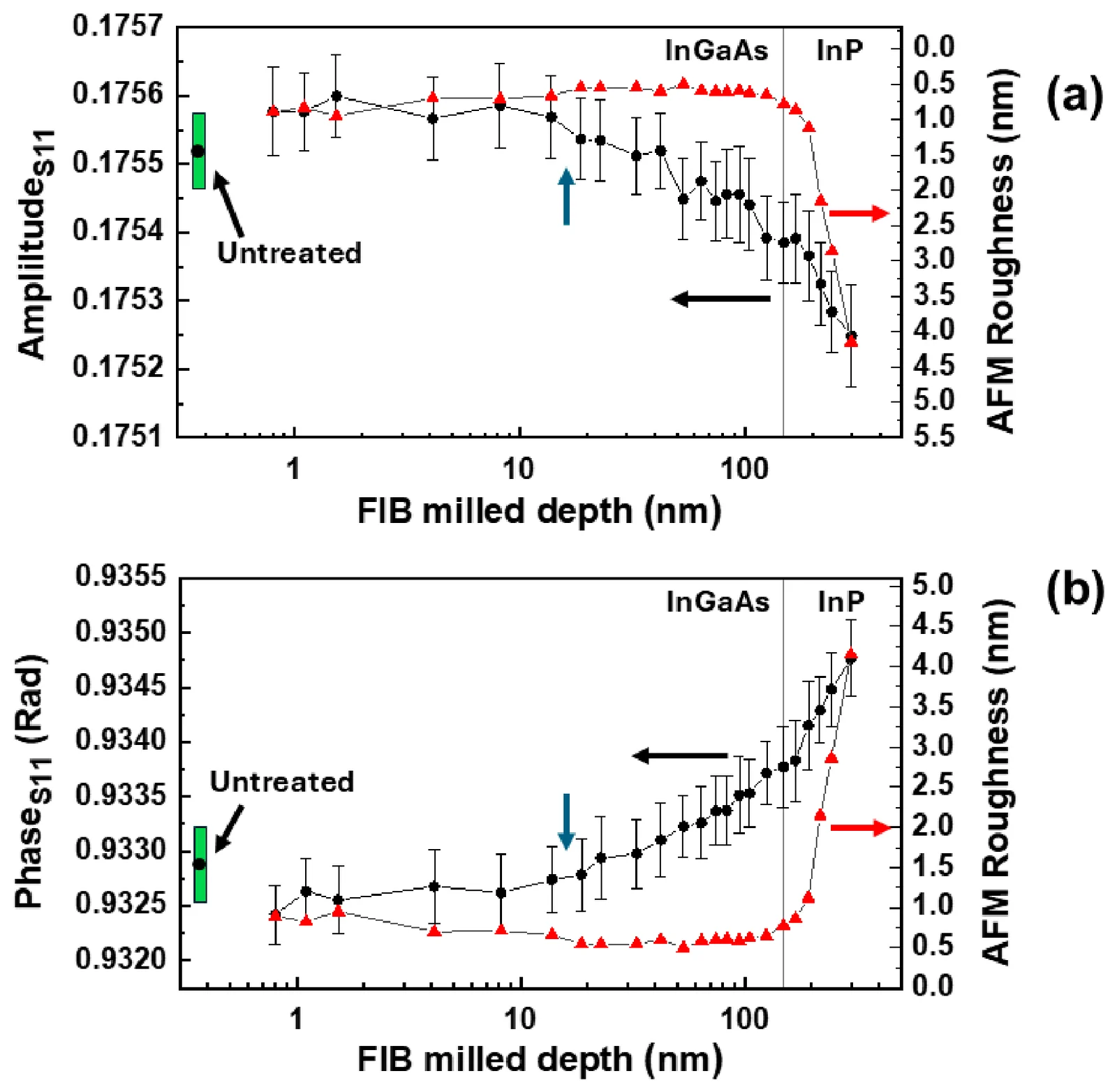
(**a**) Amplitude and (**b**) phase of S_11_ signal (black dots) for the InGaAs/InP sample upon increasing the FIB treatment ion dose, compared with surface roughness (red triangles) inside the FIB-milled areas. The vertical grey line represents the interface between InGaAs and InP layers. Blue vertical arrows indicate the FIB-milled depth limit below which the SMM signals remain nearly constant. Error bars in the plots represent the combination of standard deviation of SMM data within corresponding FIB-milled area and in unmilled regions.

**Figure 7:**
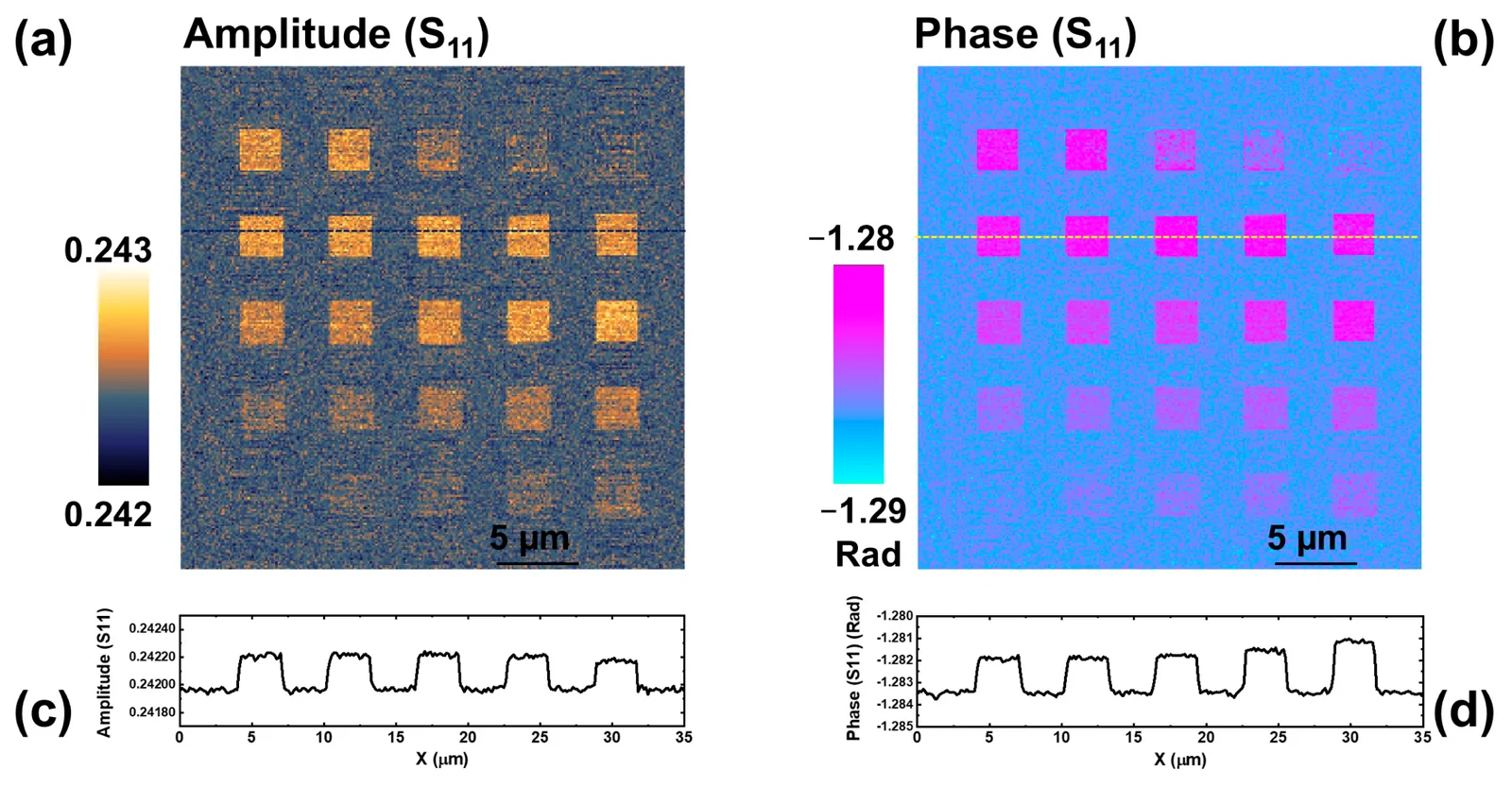
SMM data collected on the HfO_2_/InGaAs/InP sample in the dose range 1.9 × 10^14^–6.8 × 10^16^ ions/cm^2^. SMM maps of (**a**) amplitude and (**b**) phase of S_11_. Section profiles of (**c**) amplitude and (**d**) phase of S_11_ along the dashed lines shown in (**a**,**b**), respectively. SMM maps were collected at a frequency *f* = 5.483182 GHz.

**Figure 8:**
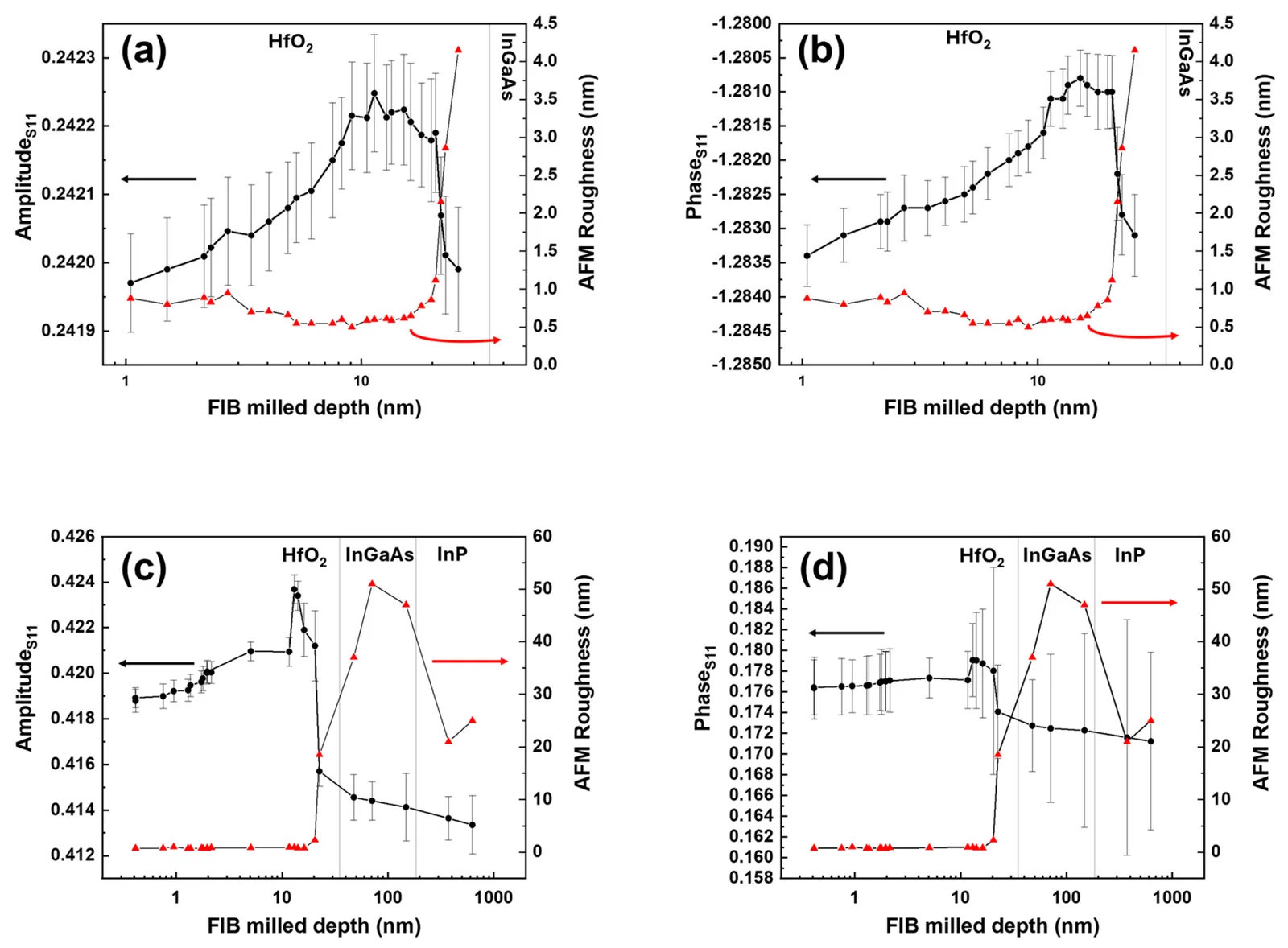
(**a**) Amplitude and (**b**) phase of S_11_ signals (black dots) for the HfO_2_/InGaAs/InP sample upon increasing the FIB treatment ion dose, compared with surface roughness (red triangles) inside the FIB-milled areas. Panels (**a**,**b**) report S_11_ data referring to the FIB patterns shown in Figure 2a. Panels (**c**,**d**) report S_11_ data in a larger FIB-milled depth range: the topmost FIB-milled depth range is shown in Figure 2b. Vertical grey lines and labels in the graphs highlight the interfaces between the HfO_2_/InGaAs and the InGaAs/InP layers. The large ion dose (i.e., FIB-milled depth) range dataset was collected using an SMM frequency *f* = 5.483425 GHz, while the lower-range dataset was acquired using a frequency *f* = 5.483182 GHz. Error bars in the plots represent standard deviation of SMM data within corresponding FIB-milled area.

In this work, the amplitude and phase of S_11_ are used as non-destructive and sensitive indicators of FIB-induced changes in the local microwave electrical response. The measured contrast may reflect the combined effects of the local electrical and dielectric environment, effective tip–sample contact conditions, sample morphology, and its structure. Reliable and unambiguous extraction of absolute impedance-related parameters would have required dedicated on-chip calibration structures and a sample-specific electromagnetic model. The analysis, therefore, focuses on possible correlations between the SMM response and the structural and morphological modifications assessed by Raman spectroscopy and AFM. Although qualitative, SMM provides complementary information on local microwave- and impedance-related variations that cannot be directly obtained from Raman or AFM measurements alone. S_11_ data collected by SMM from the FIB-treated regions of InGaAs/InP and HfO_2_ /InGaAs/InP samples are reported in Figure 5, Figure 6, Figure 7 and Figure 8. For almost all the ion doses used, the S_11_ maps (amplitude and phase) show a clear contrast between the FIB-treated areas and the outer regions (see Figure 5 and Figure 7). S_11_ amplitude and phase as a function of the FIB milling depth for the two investigated samples are reported in Figure 6 and Figure 8. The corresponding surface roughness values, measured by AFM within the FIB-treated regions, are superimposed (red data in the plots) to evaluate potential correlations between surface morphology and the SMM response, and to assess the significance of the observed S_11_ variations. Note that in Figure 6a, the roughness axis is inverted to facilitate a more direct comparison of the trend direction.

The S_11_ data for the InGaAs/InP sample are reported in Figure 5 and Figure 6. Figure 5a,b show the two-dimensional S_11_ amplitude and phase maps, in which the FIB-treated areas exhibit evident false color contrast with respect to the surrounding untreated regions, even at the lowest investigated dose of 1.96 × 10^14^ ions/cm^2^. As the FIB milling dose increases, the S_11_ amplitude data in the colored map (Figure 5a) follows a monotonic decrease (from yellow to a dark color), starting from values higher than those observed in the untreated regions. Oppositely, the phase signal map in Figure 5b shows an increase in its value, visible as a transition from blue to a dark rose color in the square FIB-treated areas. Also in this case, the phase signal starting values are different, namely smaller, than those in the untreated areas. Cross-sectional profiles of the maps corresponding to the five highest doses used are reported in Figure 5c,d, showing the S_11_ amplitude and phase behavior in the ion-irradiated square areas with respect to the outside untreated regions. At the edges of the treated areas, both signals exhibit peak-like features, which may be associated with different implantation conditions near the borders and/or artifacts due to morphological interactions of the edges of the milled structures with the SMM probe, which can be reflected in the S_11_ response. For this reason, the S_11_ data as a function of FIB-milled depth were obtained by averaging the values within the very central region of each FIB-treated square area. The S_11_ amplitude and phase data vs. FIB-milled depth, as measured with AFM, are reported in Figure 6. Relative S_11_ curves, expressed as differences with respect to the corresponding untreated regions, are also reported in the Supporting Information (Figure S3) to better highlight the magnitude of the observed variations. Starting from low ion doses, the amplitude and phase data remain almost constant up to a milling depth in the InGaAs film of about 10 nm. This behavior is supported by the fitting analysis reported in the Supporting Information (Figure S1, Tables S1 and S2). The linear fitting model in this low-dose region yields unreliable parameters, characterized by a low R^2^ coefficient of determination (labeled as R-Square in Tables S1–S5). The very low slope, affected by a large uncertainty, is consistent with a constant trend. The S_11_ amplitude and phase in this low-dose region (small FIB-milled depth) appear to be shifted with respect to the values measured in the external untreated areas (indicated by the black dots on the left side of the plots, where the green bar vertical size corresponds to the variation in experimental data). A Welch’s *t*-test confirmed a significant difference between the S_11_ mean amplitude and phase values measured in the untreated sample and in the low-milling-depth region, where the S_11_ values remain approximately constant. After a subsequent monotonically decreasing (increasing) behavior of S_11_ amplitude (phase) data, in a high-dose regime corresponding to an FIB milling depth larger than 180 nm (right part of the S_11_ plots), the S_11_ data exhibit a clear linear behavior, as confirmed by the fitting analysis (Figure S2, Tables S3 and S4). In the whole FIB-milling depth range investigated, the AFM roughness data are initially roughly constant and limited below 1 nm up to an FIB milling depth of ~180 nm, then start increasing with a linear behavior (see fitting analysis in Figure S2 and Table S5).

A reasonable interpretation of these findings is that, at these low doses, the treated regions undergo Ga implantation, which induces damage in the form of amorphization of a shallow InGaAs layer, thereby modifying its electrical properties even at low doses. While a small fraction of the film thickness is affected by implantation, the SMM interaction volume still includes a large portion of the underlying undamaged crystalline InGaAs, explaining the nearly flat behavior. This finding is consistent with the Raman data in Figure 4, where the spectra recorded up to a milling depth of ~10 nm are very similar to each other, but clearly different from the reference spectrum of the untreated areas, showing a significant broadening of the InGaAs Raman band. The limited and almost constant AFM roughness data up to a ~180 nm FIB-milled depth point to a negligible morphological effect on SMM data collection in this range. At milling depth values larger than ~10 nm, the FIB process is expected to have reached a steady-state milling and damage regime that is known to produce an amorphous layer of approximately 40 nm [10], which becomes increasingly comparable to the remaining crystalline InGaAs thickness.

In parallel, the Raman spectra show a change in the relative intensity of the InAs-like and GaAs-like bands, suggesting a modification of the In/Ga composition of the semiconductor, which further affects the electrical properties detected by SMM as a monotonic variation in the amplitude and phase data in the ~10–180 nm FIB-milled depth range in Figure 6. At milling depths around 130–150 nm, and extending to somewhat larger values, the InGaAs layer is reduced to only a few nanometers to a few tens of nanometers in thickness, or may already be fully removed. In this regime, the S_11_ response may begin to include contributions from the shallow InP region (likely damaged), although its interpretation becomes progressively more affected by the increasing roughness.

A further relevant change in the S_11_ data is observed for milling depths larger than ~180 nm, i.e., exceeding the initial thickness of the InGaAs film by approximately 30 nm. In this regime, well above the transition from the InGaAs layer to the underlying InP substrate, the S_11_ response continues to evolve smoothly. Both the amplitude and phase signals exhibit an approximately linear trend over this high-depth range, as also supported by the linear fitting analysis reported in the Supporting Information (Figure S2, Tables S3 and S4). Notably, the surface roughness remains limited to approximately 1 nm up to milling depths of about 200 nm, by which point the contribution of the InP substrate is already expected to be reflected in the S_11_ response. Over the entire investigated depth range, the RMS roughness does not exceed approximately 5 nm. Beyond 200 nm, however, it increases more markedly and exhibits an approximately linear trend (Figure S2 and Table S5 in the Supporting Information), consistent with the behavior observed in the S_11_ data. These observations suggest that the progressive S_11_ variation is compatible with the accumulation of FIB-induced damage and with changes in the structural and electrical properties of the material probed by SMM. A contribution from surface morphology cannot be excluded, particularly at higher milling depths, where the RMS roughness increases and follows an approximately linear trend. However, the limited roughness observed up to approximately 200 nm suggests that the S_11_ evolution cannot be attributed to topographical effects alone. Rather, the measured response likely reflects a combination of progressive FIB-induced material damage and morphological changes. The former interpretation is supported by the presence of defect bands, notably one with increasing intensity visible at high doses in the Raman signal of InP, whereas the other may contribute to the response through changes in the tip–sample interaction. Since these contributions cannot be quantitatively disentangled from the present uncalibrated measurements, a more definitive assessment will require dedicated investigations combining calibrated SMM with independently controlled structural and morphological modifications.

On the other hand, for the HfO_2_/InGaAs/InP sample, the presence of the three-layer stack and the different FIB milling rates of the three materials generate a more complex scenario compared to the sample consisting of InGaAs/InP.

The S_11_ data for the HfO_2_/InGaAs/InP sample are reported in Figure 7 and Figure 8. Figure 7a,b show two-dimensional maps of the S_11_ amplitude and phase, respectively, where a clear contrast between the FIB-treated areas and the surrounding untreated regions is already visible, even at the lowest ion doses in the investigated range, 1.96 × 10^14^–6.08 × 10^16^ ions/ cm^2^. S_11_ data extracted from the maps are shown in Figure 8a,b, where RMS roughness is also reported to facilitate trend comparison and assess possible correlations between the two kinds of data. As the FIB milling dose increases, both the S_11_ amplitude and phase exhibit a similar qualitative evolution, with an initial monotonically increasing variation followed by a pronounced maximum (or a plateau) at a dose of about 2 × 10^16^–3 × 10^16^ ions/ cm^2^, corresponding to a milled depth of approximately 10–15 nm, and a subsequent sharp decrease.

Comparable to the discussion for the InGaAs/InP sample, for the low-milling-depth regime, the AFM roughness remains very limited, remaining below 1 nm, and no clear one-to-one correlation with the S_11_ signals is observed up to a milling depth of about 20 nm. At a milling depth of ~20 nm, the roughness starts to increase sharply, and similarly, the S_11_ signals experience a sharp decrease. These findings indicate that for low ion doses, the SMM signals follow the structural evolution of the material, namely the onset of damage in terms of implantation and initial thinning of the HfO_2_ capping layer.

To obtain additional information and further assess the observed trends, additional FIB-patterned arrays were fabricated over an extended dose range, including both lower and higher ion doses, as described in Section 2.1. The corresponding S_11_ amplitude and phase data are reported in Figure 8c,d. Relative S_11_ curves for the four panels of Figure 8, expressed with respect to the untreated regions and comparing the two measurement runs, are reported in the Supporting Information (Figure S4). Although the measurements in Figure 8a,b were acquired at different times and with different SMM probes with respect to the data reported in Figure 8c,d, the overall qualitative trends remain consistent, despite the discrepancies in the absolute S_11_ values. These findings support the capability of the SMM technique to reproduce the qualitative behavior of the investigated materials under FIB irradiation, provided that the frequency working point is carefully set to that corresponding to the absolute minimum in S_11_ amplitude for the SMM equipment.

Taken together, and supported by AFM and Raman spectroscopy data, the curves in Figure 8 show that at the lowest doses (up to ~4 × 10^16^ ions/cm^2^), the observed modulation of the SMM signal can be ascribed primarily to ion implantation in the oxide accompanied by a progressive thinning of the HfO_2_ capping layer, which remains smaller than the initial thickness. In this regime, the S_11_ response is essentially reflecting the progressive changes in the electrical properties of HfO_2_ as a result of Ga incorporation and structural damage, which are expected to alter both its effective thickness and dielectric behavior. As confirmed by Raman spectra, which in this dose range show only limited broadening of the InGaAs-related features, the HfO_2_ layer still partially protects the underlying InGaAs film from significant damage. A slight change in the relative intensity of the InAs-like and GaAs-like Raman bands suggests that a small amount of Ga ions may already penetrate through the oxide and reach the crystalline InGaAs layer, increasing the local Ga concentration within the semiconductor matrix. However, the structural change in the InGaAs layer is expected to be quite modest. At intermediate doses, corresponding to milling depths of ~10–15 nm, the HfO_2_ layer becomes strongly modified and progressively less effective as a protective cap. In this range, where the roughness is still limited, the SMM response reaches a maximum and then begins to decrease, indicating that the microwave probe is sensing not only the damaged oxide but also the underlying semiconductor stack, given the sensing depth of the SMM technique. Raman data support this interpretation by showing a more evident broadening of the InGaAs-related spectral features, consistent with implantation-induced disorder and amorphization. Around a dose of about 3 × 10^16^ ions/ cm^2^, the Raman spectrum becomes very similar to that observed for the uncapped InGaAs/InP sample at the lowest investigated dose, suggesting that a comparable level of damage, broadening, and structural modification is eventually reached in the semiconductor beneath the oxide cap. These observations suggest that the HfO_2_ layer initially acts as an effective buffer against Ga implantation, but also that its protective role progressively weakens as ion-induced damage, thinning, and local morphological inhomogeneity develop.

For a larger milling depth, around 20 nm, the SMM signals show a sharp decrease. This variation occurs in a dose range where the surface roughness also increases markedly but still remains below ~5 nm. At a milling depth of about 20 nm, only ~15 nm of the original 35 nm thick HfO_2_ layer remains. Therefore, the SMM response is expected to include a contribution from the underlying InGaAs layer, which may have already undergone FIB-induced structural and electrical modifications. A direct correlation between the SMM response and the modifications of the underlying layers cannot be established unambiguously, since the measured S_11_ signals may be affected by morphology-related effects. Accordingly, the observed response may reflect a combination of surface morphology and FIB-induced modifications of the multi-layer structure.

For doses above 5 × 10^16^ ion/cm^2^ corresponding to FIB milling depths greater than approximately 20 nm, the surface undergoes a pronounced morphological evolution, driven by the nonuniform milling of the material and the different milling rates of the individual layers. This produces not only an increase in RMS roughness, but also large dose-dependent fluctuations, likely associated with an evolving surface topography in which regions at different height levels (corresponding to HfO_2_, InGaAs, or even InP) may coexist within the same FIB-treated area. Interestingly, in this high-dose range, the SMM signals decrease monotonically after the initial sharp change observed at an FIB-milled depth of about 20 nm, whereas the RMS roughness exhibits much larger fluctuations. The SMM response, therefore, does not simply track the roughness evolution, and it also does not show comparably large fluctuations in its associated uncertainty. This behavior is particularly evident in the S_11_ amplitude signal, for which the relative uncertainty is smaller. The absence of a direct correspondence between the SMM response and the roughness suggests that morphology alone may not fully account for the observed signal evolution, particularly over the entire range of roughness values investigated.

The complex morphology observed in this regime is likely related to the strong difference in milling rates between HfO_2_ and the underlying semiconductor layers. Since the milling rate of semiconductors is approximately 12 times higher than that of HfO_2_, small local variations in the thickness or continuity of the oxide lead to the formation of localized pits, which are subsequently amplified as the underlying InGaAs and InP are exposed and removed more rapidly. As a result, worm-like or pillar-like HfO_2_ residues may coexist with lower regions exposing the underlying semiconductor, as suggested by the SEM and AFM images in Figure 2. The resulting coexistence of different materials and height levels can generate a complex, spatially mixed SMM response.

At the same time, the monotonic decrease in the S_11_ signals may also be related to the progressive structural and compositional modification of the material probed by SMM. In particular, the sharp change occurring when the HfO_2_ layer is progressively removed may involve the transition from a dielectric cap to the underlying FIB-damaged semiconductor, together with increasing amorphization, ion incorporation, and modification of the local electrical properties. From this perspective, the observed response is compatible with a combined effect of surface morphology, local material composition, layer removal, and FIB-induced structural damage. The present measurements do not allow these contributions to be quantitatively separated. A more definitive assessment would require dedicated SMM measurements on single-layer and multi-layer structures with independently controlled morphology and well-defined structural damage. Such an investigation is beyond the scope of the present work and will be addressed in future studies.

As a whole, our SMM analysis provides a qualitative assessment of Ga^+^ FIB-induced electrical damage in capped and uncapped InGaAs/InP heterostructures, especially in a low-ion-dose regime, highlighting how the high-*k* dielectric may influence the relative contributions of implantation, amorphization, and layer removal in a technologically relevant mid-IR highly doped semiconductor platform.

## 4. Conclusions

This work shows that scanning microwave microscopy is a sensitive and non-destructive probe potentially suitable for tracking FIB-induced changes in III–V heterostructures, especially at lower irradiation doses, even without calibration or de-embedding. In the uncapped InGaAs/InP sample, the SMM response evolves from an implantation-dominated regime in the near-surface InGaAs layer toward a signal that may also be influenced by the damaged and exposed InP substrate as milling proceeds. The smooth evolution of the S_11_ signals across the InGaAs/InP interface, together with the limited surface roughness over a substantial part of this range, supports an important contribution from FIB-induced structural and electrical modifications of the material probed by SMM.

In the HfO_2_/InGaAs/InP stack, the evolution is more complex: the HfO_2_ capping layer initially modulates ion penetration, but it is progressively modified, thinned, and roughened, leading to a heterogeneous microwave response that may reflect the combined effect of structural factors, such as cap degradation, semiconductor damage, and multi-layer coexistence, and of morphological evolution. In particular, at higher milling depths, the monotonic evolution of the S_11_ signals does not directly follow the fluctuations in surface roughness, suggesting that the response cannot be attributed to topography alone. Instead, it is compatible with a coupled contribution from morphology, layer removal, local material composition, and FIB-induced structural and electrical modifications. Taken together with AFM and Raman spectroscopy, the SMM data indicate that the two stacks follow distinct damage pathways and that the microwave response is already sensitive to early-stage FIB-induced changes, especially at low doses, when morphological alterations are still limited. At higher doses, the interpretation of uncalibrated S_11_ signals becomes less straightforward since several effects may coexist, including surface roughening, non-uniform layer removal, exposure of different materials, and progressive structural damage. Nevertheless, the combined evolution of the SMM, AFM, and Raman signals provides evidence that the measured microwave contrast is not governed by morphology alone and may also contain information on the FIB-modified material structure.

Overall, the results support SMM as a promising qualitative diagnostic tool for evaluating FIB processing windows in layered semiconductor systems relevant to mid-infrared nanophotonics. Nevertheless, robust quantitative use of this approach will require on-chip calibration and de-embedding structures, together with a measurement protocol and sample-specific models that account explicitly for morphology-induced effects and the heterogeneous nature of multi-layer structures.

More broadly, the combined SMM–AFM–Raman methodology framework is transferable to other doped semiconductor/dielectric stacks and to different FIB-processing conditions where correlated structural and electrical damage must be assessed.

## Data Availability

The original data presented in the study are openly available in ZENODO at https://doi.org/10.5281/zenodo.21027153.

## Supplementary Materials

The following supporting information can be downloaded at: https://www.mdpi.com/article/10.3390/nano16161042/s1, Figure S1: S_11_ amplitude and phase versus ion dose, with linear fits; Figure S2: S_11_ amplitude and phase, and AFM roughness, versus FIB-milled depth for the InGaAs/InP sample, with linear fits; Figure S3: Relative S_11_ amplitude and phase changes versus FIB-milled depth for the InGaAs/InP sample; Figure S4: Relative S_11_ amplitude and phase changes versus FIB-milled depth for the HfO_2_/InGaAs/InP sample; Table S1: Linear fit parameters for S_11_ amplitude at low ion doses; Table S2: Linear fit parameters for S_11_ phase at low ion doses; Table S3: Linear fit parameters for S_11_ amplitude at high milled depths; Table S4: Linear fit parameters for S_11_ phase at high milled depths; Table S5: Linear fit parameters for AFM roughness at high milled depths.
