# Supplementary material for: A Scanning Microwave Microscopy Study of FIB-Induced Local S11 Response Changes in InGaAs/InP and HfO2/InGaAs/InP Heterostructures
Source: Nanomaterials (Basel). 2026 Aug 21;16(16):1042. doi: 10.3390/nano16161042 (PMC13516979; doi:10.3390/nano16161042)
Supplement: Supplementary file 1 [file nanomaterials-16-01042-s001.zip › nanomaterials-4456241-supplementary.pdf]

SUPPORTING INFORMATION

A Scanning Microwave Microscopy Study of FIB-Induced Local S_{11} Response Changes in InGaAs/InP and HfO_2/InGaAs/InP Heterostructures

Linear fitting outputs for the InGaAs/InP sample: S_{11} data and AFM roughness data

1) Fitting at low values of ion dose (milled depth)

Fitting procedure was performed to assess the possible constant behavior at low ione doses (small milled depth), corresponding to an ion dose interval 2.0×10^{14} - 5.7×10^{15} ions/cm^2, corresponding to milled depth interval from -0.65 nm to 13.8 nm.

We reported here the plot and performed the fitting using the S_{11} signals vs. ion dose datasets (in place of signals versus milled depth, as in the main text), since the first negative values of depth cannot be visualized in Log scale. Since our aim is to assess the flatness of the behavior, having more data points for the evaluation increases the significance of the test.

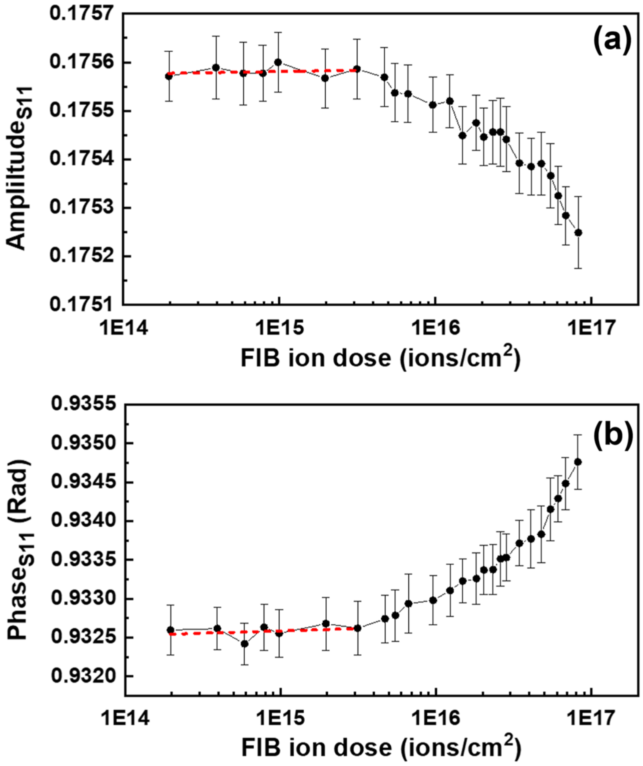

Figure S1: S_{11} (a) amplitude and (b) phase data reported versus ion dose. The dashed red segment represents the linear fitting plot in the investigated data interval.

Equation	y = a + bx
Intercept (a)	0.1756 ± 1.43064E-4
Slope (b)	-1.25258E-6 ± 9.54863E-6
Residual Sum of Squares	0.24603
R-Square (COD)	**0.00286**
Adj. R-Square	-0.16333

Table S1: Intercept and slope of the linear fitting evaluated on the S_{11} amplitude data for InGaAs/InP sample. The small R Square coefficient and the low value of the slope indicate an almost constant value for the S_{11} amplitude in the investigated interval.

Equation	y = a + bx
Intercept (a)	0.93087 ± 0.00117
Slope (b)	1.15703E-4 ± 7.82918E-5
Residual Sum of Squares	0.56473
R-Square (COD)	**0.26687**
Adj. R-Square	0.14468

Table S2: Intercept and slope of the linear fitting evaluated on the S_{11} phase data for InGaAs/InP sample. The small R Square coefficient and the low value of the slope indicate an almost constant value for the S11 phase in the investigated interval.

2) *Fitting at high values of ion dose (milled depth)*

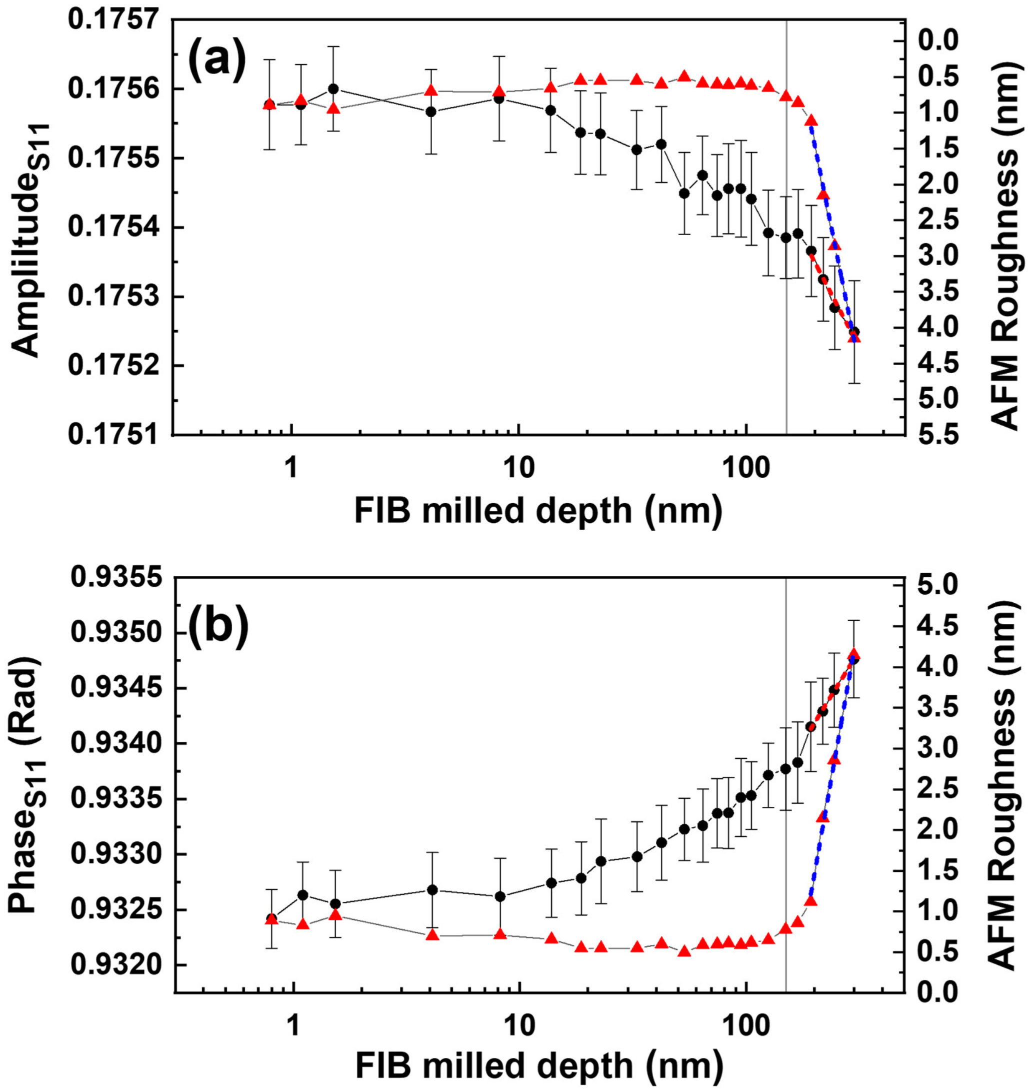

Figure S2: S_{11} (a) amplitude and (b) phase data for InGaAs/InP sample reported versus FIB milled depth, with superimposed AFM roughness data. The dashed red and blue segments represent the linear fitting plots in the investigated data interval. The vertical grey line marks the interface between the two layers of the heterostructure. The circle and triangle symbols correspond to the S11 and roughness data, respectively.

S11 data fitting

Equation	y = a + bx
Intercept (a)	0.17676 ± 1.15335E-4
Slope (b)	-6.12344E-4 ± 4.92926E-5
Residual Sum of Squares	0.05666
R-Square (COD)	**0.98093**
Adj. R-Square	0.97457

Table S3: Intercept and slope of the linear fitting evaluated on the S_{11} amplitude data for InGaAs/InP sample in the milled depth interval from 170 nm to 300 nm.

Equation	y = a + bx
Intercept (a)	-0.00709 ± 5.9907E-4
Slope (b)	0.00365 ± 2.55482E-4
Residual Sum of Squares	7.10121E-9
Pearson's r	0.99274
R-Square (COD)	**0.98553**
Adj. R-Square	0.9807

Table S4: Intercept and slope of the linear fitting evaluated on the S_{11} phase data for InGaAs/InP sample in the milled depth interval from 170 nm to 300 nm.

AFM Roughness data fitting

Equation	y = a + bx
Intercept (a)	-34.68617 ± 1.79504
Slope (b)	15.70548 ± 0.75638
Residual Sum of Squares	0.02244
R-Square (COD)	**0.99538**
Adj. R-Square	0.99307

Table S5: Intercept and slope of the linear fitting evaluated on the AFM Roughness data for InGaAs/InP sample in the milled depth interval from 170 nm to 300 nm.

Relative variation of S_{11} signals

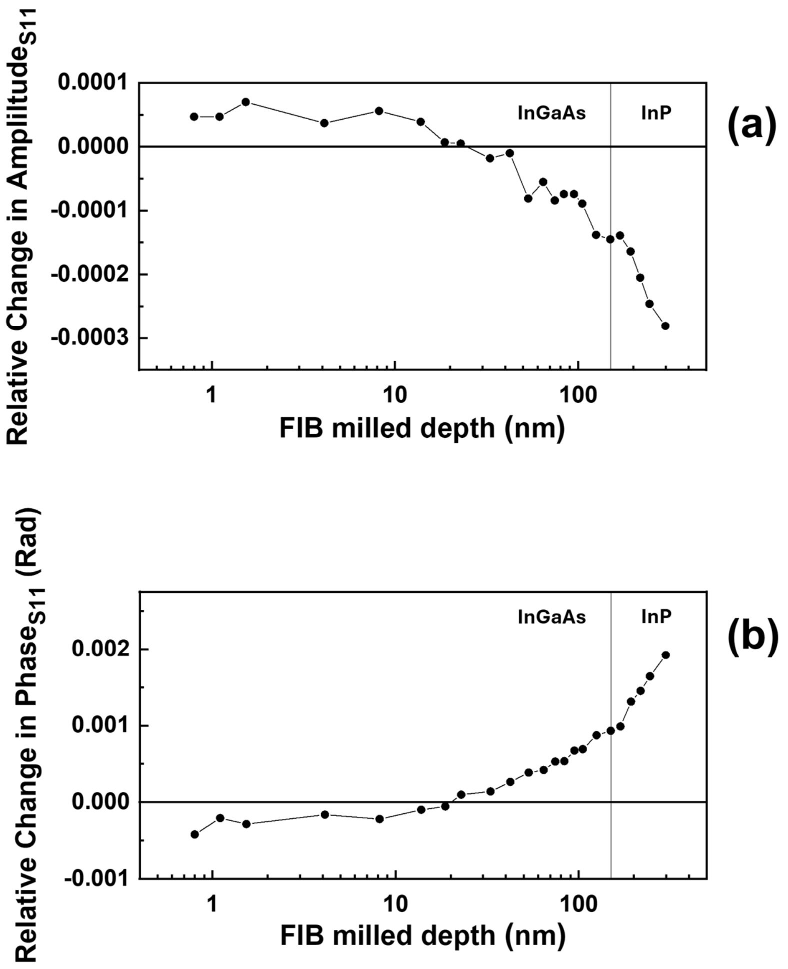

Figure S3: Relative (a) amplitude and (b) phase changes in S_{11} signals with respect to the untreated regions, reported versus FIB milled depth for InGaAs/InP sample. For better readability, data are represented without error bars which can be found in Figure 6 of the manuscript. The zero level of S_{11} data corresponds to the outside untreated areas. The vertical line indicates the interface between InGaAs layer and the underlying InP substrate.

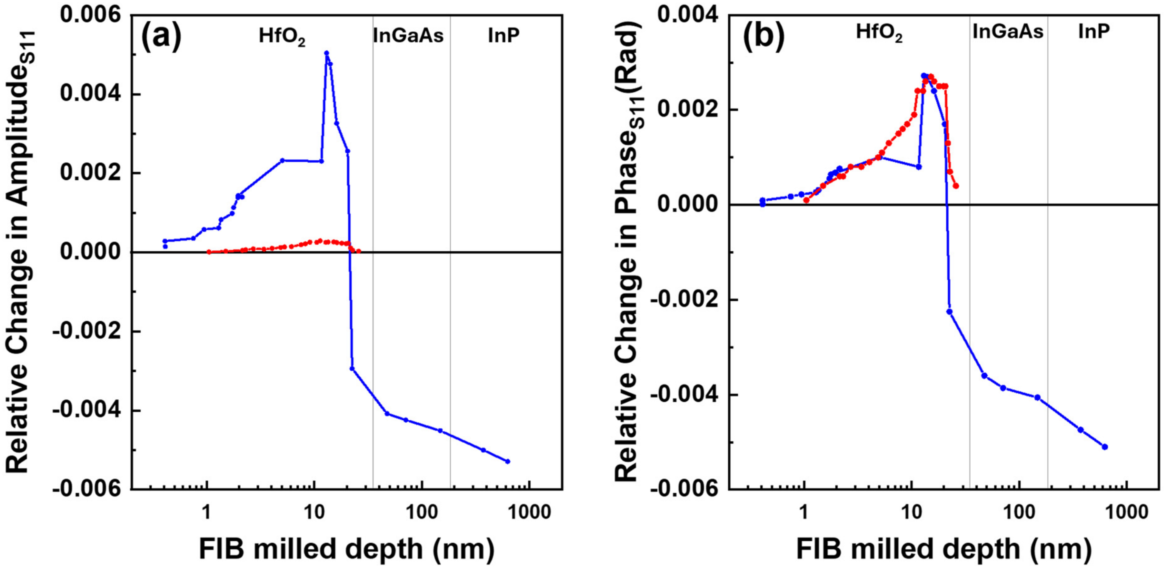

Figure S4: Comparison between relative (a) amplitude and (b) phase changes in S_{11} signals versus FIB milled depth for the FIB-treated regions on HfO_2/InGaAs/InP sample. Data are relative to the two measurement runs shown in Figure 8. For better readability, data are represented without error bars which can be found in Figure 8 of the main text. The data shown in blue and red correspond to FIB treatments in the wider and narrower dose ranges, respectively. The vertical lines indicate the interfaces between the layers, as labeled inside the plot.
